# BarTeL, a Genetically Versatile, Bioluminescent and Granule Neuron Precursor-Targeted Mouse Model for Medulloblastoma

**DOI:** 10.1371/journal.pone.0156907

**Published:** 2016-06-16

**Authors:** Gregory M. Shackleford, Xiang-He Shi, Kimberly S. Swanson, Min Y. Mahdi, Ignacio Gonzalez-Gomez, Shahab Asgharzadeh, Massimo D’Apuzzo, Anat Erdreich-Epstein, Rex A. Moats

**Affiliations:** 1 Division of Hematology, Oncology and Blood & Marrow Transplantation, Department of Pediatrics, The Saban Research Institute, Children’s Hospital Los Angeles, and Keck School of Medicine, University of Southern California, Los Angeles, California, United States of America; 2 Department of Molecular Microbiology and Immunology, Keck School of Medicine, University of Southern California, Los Angeles, California, United States of America; 3 Department of Radiology, The Saban Research Institute, Children’s Hospital Los Angeles, and Keck School of Medicine, University of Southern California, Los Angeles, California, United States of America; 4 Department of Pathology, Keck School of Medicine, University of Southern California, Los Angeles, California, United States of America; 5 Department of Biomedical Engineering, University of Southern California, Los Angeles, California, United States of America; 6 Department of Pathology, City of Hope National Medical Center, Duarte, California, United States of America; University Hospital of Navarra, SPAIN

## Abstract

Medulloblastomas are the most common malignant pediatric brain tumor and have been divided into four major molecular subgroups. Animal models that mimic the principal molecular aberrations of these subgroups will be important tools for preclinical studies and allow greater understanding of medulloblastoma biology. We report a new transgenic model of medulloblastoma that possesses a unique combination of desirable characteristics including, among others, the ability to incorporate multiple and variable genes of choice and to produce bioluminescent tumors from a limited number of somatic cells within a normal cellular environment. This model, termed BarTeL, utilizes a *Bar**hl1* homeobox gene promoter to target expression of a bicistronic transgene encoding both the avian retroviral receptor TVA and an eGFP-Luciferase fusion protein to neonatal cerebellar granule neuron precursor (cGNP) cells, which are cells of origin for the sonic hedgehog (SHH) subgroup of human medulloblastomas. The *Barhl1* promoter-driven transgene is expressed strongly in mammalian cGNPs and weakly or not at all in mature granule neurons. We efficiently induced bioluminescent medulloblastomas expressing eGFP-luciferase in BarTeL mice by infection of a limited number of somatic cGNPs with avian retroviral vectors encoding the active N-terminal fragment of SHH and a stabilized MYCN mutant. Detection and quantification of the increasing bioluminescence of growing tumors in young BarTeL mice was facilitated by the declining bioluminescence of their uninfected maturing cGNPs. Inclusion of eGFP in the transgene allowed enriched sorting of cGNPs from neonatal cerebella. Use of a single bicistronic avian vector simultaneously expressing both *Shh* and *Mycn* oncogenes increased the medulloblastoma incidence and aggressiveness compared to mixed virus infections. Bioluminescent tumors could also be produced by *ex vivo* transduction of neonatal BarTeL cerebellar cells by avian retroviruses and subsequent implantation into nontransgenic cerebella. Thus, BarTeL mice provide a versatile model with opportunities for use in medulloblastoma biology and therapeutics.

## Introduction

Medulloblastomas are primitive neuroectodermal brain tumors and the most common malignant brain tumor of childhood [1, 2]. Despite aggressive multi-modality therapy, cure rates in patients with high-risk disease remain poor. Moreover, serious sequelae such as cognitive decline and hearing loss impair many survivors’ subsequent quality of life [1, 2].

Current consensus divides medulloblastomas into four major molecular subgroups: subgroup with activated WNT pathway, subgroup with activated sonic hedgehog (SHH) pathway, and Groups 3 and 4 [3–7]. Whereas the cell of origin for Group 4 medulloblastomas is not conclusively known, and the WNT tumors are derived from lower rhombic progenitor cells, the SHH subgroup have been shown to originate from cerebellar granule neuron precursors (cGNPs) [8–11]. During development, cGNPs migrate rostrally from the rhombic lip of the embryonic hindbrain to form the external granule layer (EGL) of the developing cerebellum, a region in the brain responsible for motor control and spatial orientation [12]. In neonates, the proliferative cGNPs of the outer layer of the EGL stop proliferating and migrate inward to form the internal granule layer (IGL). During this process, the cGNPs differentiate to give rise to the granule cell neurons of the IGL.

An important approach to understanding the molecular interactions governing medulloblastoma formation, growth and metastasis is through the use of genetically-engineered mice that develop medulloblastomas. The typical engineered mouse requires generation of a separate transgenic or knockout line for each gene studied, thus requiring crossbreeding to study genetic interactions. Additionally, the mice generally express the oncogenic transgene or knock-out construct of interest in all or most cells in which the expression-controlling promoter is normally expressed, which is uncharacteristic of most human tumors save those involving germline mutations. Moreover, the mice are often not designed to facilitate imaging of tumor development and regression. Some of these disadvantages can be alleviated with the use of *Tva* transgenics, in which the avian receptor for subgroup A avian sarcoma-leukosis retroviruses, TVA, is produced via a cell type-specific promoter to allow individual somatic cell susceptibility to avian viral vector transduction [13–15].

Here we report a novel bicistronic Barhl1-TVA-eGFP/luciferase transgenic mouse model for medulloblastoma. In this model, a *Tva* transgene driven by the *Barhl1* (BarH-like homeobox 1) promoter is transiently expressed in the cGNP cells of newborn mice, enabling infection of a limited number of these cells with oncogene-carrying avian viral vectors to generate medulloblastomas. Among other advantages, a unique feature of this model is the transient co-expression of TVA with an eGFP/luciferase fusion protein in these medulloblastoma cells of origin, which allows *in vivo* bioluminescent detection and monitoring of medulloblastomas as they arise on a decreasing background. We demonstrate the utility of this model both *in vivo* and *ex vivo* using the *Shh* and *Mycn* oncogenes and describe the use of a potent bicistronic avian retroviral vector for the production of medulloblastomas at high incidence. We anticipate that this model will be useful in studies of the biology and therapy of medulloblastoma and in studies of cerebellar development.

## Materials and Methods

### Cell lines

The following cell lines were generously provided by D.D. Bigner (Duke University): D425MED [16], D487MED [17], D283MED [18], D341MED [19], D645MG [20], and D54MG [21]. The cell lines CHLA-259 (medulloblastoma) and CHLA-266 (atypical teratoid/rhabdoid tumor) were provided by C.P. Reynolds (Texas Tech University) [22]. The following cells were purchased from the American Type Culture Collection: DF-1 (CRL-12203), QT6 (CRL-1708) and L cells (CRL-2648). HEK-293T and NIH-3T3 cells were provided by D. Kohn (UCLA) [23] and R. Weinberg (MIT) [24], respectively. Cells were from vials frozen at the time of receipt and were cultured no more than three months continuously.

### Reverse Transcription PCR (RT-PCR)

RNAs were isolated with RNeasy (Qiagen). First strand synthesis was performed using Omniscript (Qiagen). RT-PCRs were performed as previously described [25]. The number of PCR cycles used is provided in the figure legends. Primers used in RT-PCRs are listed in S2 Table.

### Transgene construction

The transgene consists of six components in the following order, from 5' to 3': *Barhl1* promoter, quail *Tva* cDNA, an internal ribosome entry site (IRES) from the equine encephalomyocarditis virus (EMCV), cDNA encoding a fusion protein of the enhanced green fluorescent protein (eGFP) and firefly luciferase, an intron from the mouse *Npm3* gene, and an SV40 polyadenylation signal sequence. We amplified the *Barhl1*, *Tva*, IRES, and *Npm3* intron components by PCR using either PfuUltra (Stratagene) or Phusion DNA polymerase (New England BioLabs), and sequences were verified before use. The *Barhl1* promoter fragment was amplified from genomic DNA of a mouse with a C57BL/6 and DBA/2 mixed-strain background. This fragment extends from 320 bp upstream to approximately 4.7 kb upstream of the BARHL1 initiation codon and contains the first 140 bp of leader sequence from the known *Barhl1* mRNA. The short variant of the *Tva* cDNA, encoding 120 amino acids (GenBank cDNA accession number L22752), was amplified from the RNA of quail QT6 cells using a 5’ primer that included an improved Kozak initiation sequence. The EMCV IRES sequence was amplified from pMIRB (gift of David Ornitz, Washington University, St. Louis) and converted by PCR to the more efficient wild-type EMCV IRES sequence described by Martin *et al*. [26]. An eGFP-luciferase fusion gene (formerly sold by Clontech), which we modified slightly to contain a flexible linker sequence between eGFP and luciferase, was included in the transgene. To potentially improve expression of the transgene, we included an intron; here we used the third intron from the mouse *Npm3* gene. All of these components were cloned into a deleted version of pGL3 (Promega), which provided only the SV40 polyadenylation sequence to the final transgene.

### Luciferase Assay

The *Barhl1* promoter fragment was cloned into the pGL3 luciferase reporter plasmid (Promega) and co-transfected with the *Renilla* luciferase control plasmid pRL-SV40 (Promega) into cell lines. Cell extracts were prepared 36 h after transfection using the Dual-Luciferase Reporter Assay System (Promega) according to the manufacturer's instructions, and firefly and *Renilla* luminescence was measured with a luminometer.

### Mice and bioluminescence imaging

The transgenic mice were generated by the University of Southern California Transgenic Core Facility, which injected our purified transgene fragment into fertilized B6D2F1 mouse eggs. This facility obtained their mice from The Jackson Laboratory. Mice were housed at The Saban Research Institute of Children's Hospital Los Angeles. Both animal facilities are accredited by the Association for Assessment and Accreditation of Laboratory Animal Care International. All mouse procedures were specifically approved by the Children’s Hospital Los Angeles Institutional Animal Care and Use Committee (protocol number 190) and were performed in strict accordance with recommendations of the latest (eighth) edition of the *Guide for the Care and Use of Laboratory Animals*. Mice were observed daily by animal facility personnel and laboratory personnel, all of whom are trained to recognize symptoms requiring euthansia. All efforts were made to alleviate any potential animal discomfort. Anesthesia was provided by isoflurane inhalation. Euthanasia was performed on any mouse showing signs of tumor or illness including head tilt or other neurological deficits, hydrocephalus, abnormal posture or movement, lethargy, rough coat, abnormal breathing, or weight loss. Endpoints for euthanasia and the cranial localization of medulloblastoma tumors precluded their size from exceeding the currently recommended limits for tumor size in mice. Euthansia was performed by CO_2_ inhalation until narcosis or apparent death, using the gradual displacement method with a flow meter as advised by *The American Veterinary Medical Association Guidelines for the Euthanasia of Animals*: *2013 Edition*, followed by cervical dislocation. When harvesting tissues for histology, euthansia was performed by isoflurane inhalation until mice were deeply anesthetized followed by perfusion with normal saline. Neonatal mouse euthansia was performed by isoflurane or CO_2_ inhalation until narcosis followed by decapitation. For ex vivo injections into cerebella of B6D2F1 mice (purchased from The Jackson Laboratory), analgesia was provided by ketoprofen prior to injection and by ibuprofen in drinking water after injection. Bioluminescence imaging (Xenogen IVIS^®^ 100) of mice was performed under isoflurane anesthesia after an intraperitoneal injection of luciferin (25 or 75 mg/kg body weight; Promega). Bioluminescence (radiance) is presented in figures as photons/sec/cm^2^/steradian.

### Retroviral constructs

Plasmid pRCASBP(A)-AP [27], containing a human placental alkaline phosphatase cDNA cloned into the ClaI site of pRCASBP(A), was provided by C. Cepko (Harvard Univ.) and used to confirm the function of our isolated quail *Tva* cDNA (S1 Fig) prior to construction of the transgene (see legend to S1 Fig for experimental details). Alkaline phosphatase-catalyzed staining of infected cells was performed as previously described [28]. The replication competent RCASBP(A) vector has been described [29, 30]. To construct RCAS-*ShhN*, a cDNA encoding the N-terminal portion of the mouse SHH protein ending with Gly-198 was amplified by PCR from pRK5-ShhN [31], a gift of P. Beachy (Stanford Univ.) via C.-M. Fan (Carnegie Institution, Baltimore, MD), and ligated into the ClaI site of pRCASBP(A). To construct RCAS-*Mycn*^*T50A*,*S54A*^, the T50A and S54A mutations were first inserted into the shorter 454-amino acid variant of mouse *Mycn* cDNA by overlap extension PCR. *Mycn* cDNA was provided by J.S.D. Chan and S.-L. Zhang (Centre Hospitalier de l'Université de Montréal, Montréal, Canada). This mutant fragment was cloned into the BamHI and EcoRV sites of pCMV-3Tag-7 (Stratagene) to attach three copies of the human MYC epitope tag to its N-terminus. This tagged version of the mutant *Mycn* was then cloned into RCASBP(A). In later experiments involving the bicistronic vector and its single-gene derivatives, we used the RCASBP(A)ΔF1’ vector, which lacks duplicated viral sequences in the plasmid outside of the viral genome itself; this vector plasmid was kindly provided by Stephen Hughes and Andrea Ferris (NIH). We modified RCASBP(A)ΔF1’ by adding AscI and SgrAI restriction sites adjacent to the ClaI cloning site of this vector, which is just upstream of a unique MluI cloning site; the modified vector is named pRCASBP(A)ΔF1’-CASM. Into this modified vector, we cloned a *Mycn*^*T58A*^ cDNA tagged at the N-terminus with 3 copies of the human MYC epitope tag. The *Mycn*^*T58A*^ cDNA was produced by overlap extension PCR and encodes the longer 462-amino acid isoform of mouse MYCN. To produce the bicistronic vector, the IRES from the transgene was fused to mouse *ShhN* by overlap extension PCR and cloned into RCASBP(A)ΔF1’-*Mycn*^*T58A*^ downstream and adjacent to *Mycn*^*T58A*^. To produce RCASBP(A)ΔF1’-*ShhN*, *Mycn*^*T58A*^ was deleted from this bicistronic vector. All PCR products were amplified using either PfuUltra (Stratagene) or Phusion DNA polymerase (New England Biolabs), and sequences were verified before use.

### Virus production and injection into mouse cerebella

Chicken DF-1 cells were transfected with individual RCAS retroviral constructs, and virus was allowed to spread throughout the culture. The *ex vivo* experiments were performed by mixing virus-containing DF-1 cell supernatants from such cultures. For mouse infection experiments in which a mixture of RCAS-*ShhN* and RCAS-*Mycn*^*T50A*,*S54A*^ were required, we co-transfected DF-1 cells with the two RCAS plasmids and a drug resistance plasmid and isolated a single colony of DF-1 cells producing both viruses. For infection of mouse cerebella with these vectors, DF-1 cells producing a single virus or both viruses were harvested, pelleted and 2 μl of the undiluted cell pellet (~2 × 10^5^ cells) were injected into each side of the cerebellum of 1–3-day-old BarTeL transgenic pups. For the production of the bicistronic virus, the vector plasmid was transiently transfected into DF-1 cells and resulting virus allowed to spread. Two μl of the cell pellet were injected into the right side only of the cerebella of BarTeL pups at 1–3 days of age. For production of the single-gene viruses derived from the bicistronic vector, a similar procedure was used with each single-gene vector being transiently transfected into different cultures and, after spread of the virus, equal numbers of cells were mixed and pelleted before injection of two μl into pup cerebella. Mice were judged to have tumors by histological examination and/or by consistent bioluminescent radiance signals at least one log above background. Histological findings and radiance signals agreed well when both were obtained.

### *Ex vivo* infection of dissociated cerebellar cells and implantation

Cerebella were dissected from euthanized two-day-old BarTeL mouse pups, and the meninges and choroid plexus were removed. They were then dissociated with papain and DNaseI with trituration as described [32, 33]. Undigested tissue chunks were allowed to settle and suspended cells were plated in Neurobasal medium with B27, 2 mM L-glutamine, 1 mM sodium pyruvate (all from Invitrogen), 1x ITS (insulin, transferrin, selenium; Sigma), and 250 ng/ml of recombinant mouse SHH protein N-terminal fragment (C25II; R&D Systems, Inc.). Freshly harvested unconcentrated RCAS virus stocks were added to the cells at 10% of final volumes of media and incubated for 4 h at 37°C. Cells were then centrifuged, and 2 μl were injected into the cerebella of 3-day-old B6D2F1 pups. Mice were imaged for bioluminescence weekly until 13 weeks of age. To check for evidence of infection in cell culture, a small aliquot of cells was removed after the 4 h infection period and prior to centrifugation, placed into a second well with fresh medium without virus and incubated at 37°C for 24 h, at which time the cells were harvested for genomic DNA preparation and PCR to check for evidence of infection. PCRs to detect the *Mycn-* and *ShhN*-containing proviruses were performed with an RCAS-specific primer and a gene-specific primer.

### Flow cytometry analysis

Cerebellar cells were dissociated as described above, filtered through a 70-micron filter, and FCS was added to 20% v/v. Flow cytometry analysis was conducted using a BD LSR II (BD Biosciences, San Jose, CA). Data were acquired and analyzed with BD FACSDiva software (San Jose, CA).

### Immunohistochemistry

Mice were perfused with PBS and brains were formalin-fixed and paraffin-embedded. In some instances mice were not perfused. Unless otherwise stated, sections were stained with the following antibodies and dilutions: anti-human MYC mAb 9E10, 1:100 (Santa Cruz Biotechnology); anti-luciferase, 1:100 (Fitzgerald Industries); anti-GFP, 1:100 (Abcam); anti-sonic hedgehog, 1:50 (Millipore cat#06–1106); anti-PGP9.5, 1:400 (Accurate Chemical & Scientific Corp.); anti-synaptophysin, pre-diluted ready-to-use (Cell Marque).

### Molecular subgrouping of BarTeL *ShhN/Mycn*^*T58A*^ medulloblastomas

Gene expression data from BarTeL *ShhN*/*Mycn*^*T58A*^ medulloblastomas and normal littermate cerebella were obtained using the Illumina MouseRef-8 v2.0 Expression BeadChip Kit. Expression data from human medulloblastomas was obtained using the Affymetrix GeneChip Human Genome U133 Plus 2.0 Array. To compare the mouse medulloblastomas to human medulloblastoma groupings, we used normal cerebellum data from each species to normalize the data by subtracting the average of the normal cerebella data from the log2 expression values of tumor samples. The human cerebella data was obtained from Gene Expression Omnibus repository (GSE4036). For the mouse samples, microarray analyses of BarTeL mouse medulloblastomas induced by bicistronic RCAS-*ShhN/Mycn*^*T58A*^ infections were compared to normal littermate cerebella. Clustering based on the first two principle component (PC) analysis of a subset of highly variable genes among human medulloblastomas (n = 631 genes [34]) was used to determine the similarity of the mouse medulloblastomas to subgroups of human medulloblastomas.

## Results

### Identification and characterization of the *Barhl1* promoter for transgene expression in cGNP cells

To identify a gene whose promoter functions with high specificity in medulloblastoma and, presumably, in medulloblastoma cells of origin, we utilized the Serial Analysis of Gene Expression (SAGE) database of the Cancer Genome Anatomy Project [35]. We used the human SAGE Digital Gene Expression Displayer at this site to query the SAGE database for genes with medulloblastoma-specific expression. This analysis identified *BARHL1* as a gene exhibiting high average expression in 14 of 20 medulloblastoma libraries. Only one of the 166 non-cerebellar tumor or tissue libraries expressed *BARHL1*, and that was at the lowest detectable level (S1 Table). Thus, this database query gave us our first indication that the mouse or human *BARHL1* promoter might be appropriate for use in a new transgenic model of medulloblastoma. Further support is provided by reports that *Barhl1* is expressed in the cGNPs of the perinatal EGL [36, 37], which are known cells of origin for some medulloblastomas [10, 11, 38, 39].

**Fig 1:**
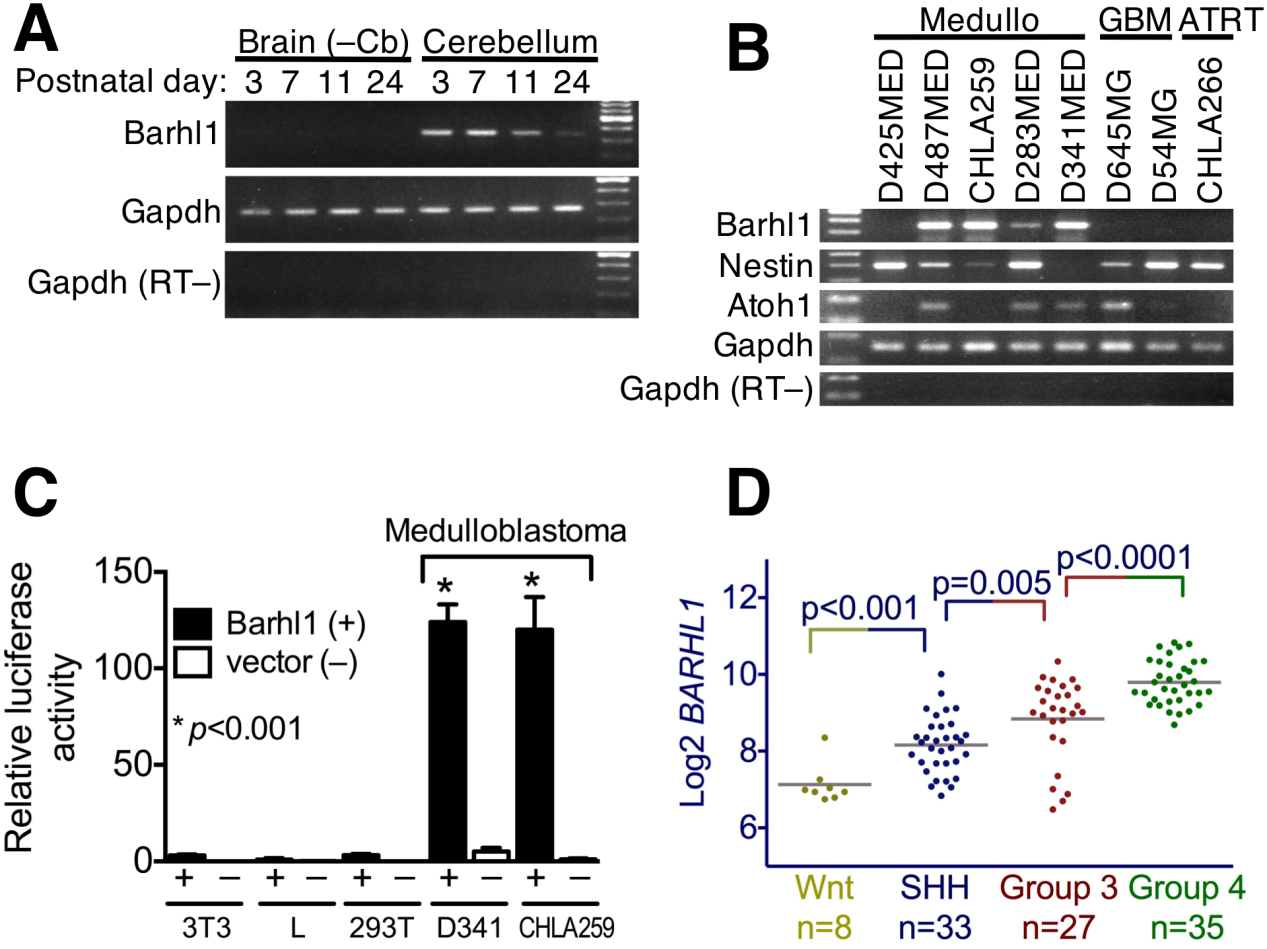
*Barhl1* is expressed in cerebellum of neonatal mice and medulloblastoma cell lines, and an isolated *Barhl1* promoter is active in medulloblastoma cell lines. (A) RT-PCR analysis of *Barhl1* expression in mouse cerebellum and non-cerebellar brain tissue at postnatal days 3, 7, 11 and 24. The PCR cycle number used was limited for *Barhl1* (32 cycles) and *Gapdh* (23 cycles) in order to enable distinction of RNA levels in cerebellum samples; high cycle number (40 cycles) showed only a faint *Barhl1* product in non-cerebellum lanes. The last lane contains 100-bp markers. PCR Primers are listed in S2 Table. (B) RT-PCR analysis of the expression of *BARHL1*, *NESTIN* and *ATOH1* in human medulloblastoma, glioblastoma and atypical teratoid/rhabdoid tumor cell lines. All PCRs were 40 cycles except *GAPDH* (23 cycles). PCR Primers are listed in S2 Table. (C) Expression of *BARHL1* in the four subgroups of human medulloblastoma. *BARHL1* gene expression data were obtained from the R2 genomic analysis and visualization platform and plotted according to subgroup. The database used was “Tumor Medulloblastoma (core transcript)–Northcott– 103 –rma_sketch–huex10t”. (D) Testing the *Barhl1* promoter in a luciferase reporter assay for activity in medulloblastoma cell lines. The Barhl1-luciferase reporter plasmid was co-transfected with a constitutive *Renilla* luciferase control plasmid into the cell lines shown. Data were corrected for transfection efficiency. The first lane contains 100-bp markers.

Given the evidence of *BARHL1* expression in cGNPs and its specificity for medulloblastoma tumors, we therefore examined its expression in mouse brains and in human brain tumor cell lines. RT-PCRs of RNAs of brains from young mice showed strong *Barhl1* expression in the cerebellum in the first week of life, which decreased to low levels by postnatal day 24 (Fig 1A). The temporal decline in *Barhl1* expression that we observed is consistent with that seen by others as the cGNPs differentiate and migrate from the EGL to the IGL during the first few postnatal weeks [36, 37, 40–42].

In a survey of human brain tumor cell lines, we found *BARHL1* expression only in the medulloblastoma cell lines (four of five examined) and not in the glioblastoma or atypical teratoid/rhabdoid tumor cell lines (Fig 1B). These expression data are in good agreement with the SAGE analysis described above. *NESTIN* and *ATOH1* were expressed in some medulloblastoma cell lines but also in other tumor cell lines.

We examined the expression of *BARHL1* in the four subgroups of human medulloblastomas by querying the publicly-available databases of Northcott *et al*. and Kool *et al*. [4, 6] in the R2 Genomics Analysis and Visualization Platform [43]. These analyses showed that the relative levels of *BARHL1* expression in subgroups are as follows: Group 4 > Group 3 > SHH > WNT (Fig 1C and S4 Fig).

To identify a *Barhl1* promoter, we first isolated a 4.4-kb genomic DNA fragment from the 5’ end of the mouse *Barhl1* gene. We then tested it for promoter activity in two of the human medulloblastoma cell lines *versus* other lines using a luciferase reporter plasmid (Fig 1D). This *Barhl1* promoter fragment drove strong luciferase expression in the medulloblastoma cells, but not in other lines, suggesting its specificity and activity in medulloblastoma. Together, the results suggest that the *Barhl1* promoter fragment would be appropriate for use in our new transgenic mouse model.

### Characterization of a *Barhl1* promoter-driven, genetically versatile, bioluminescent and cGNP-targeted transgenic mouse model for medulloblastoma

**Fig 2:**
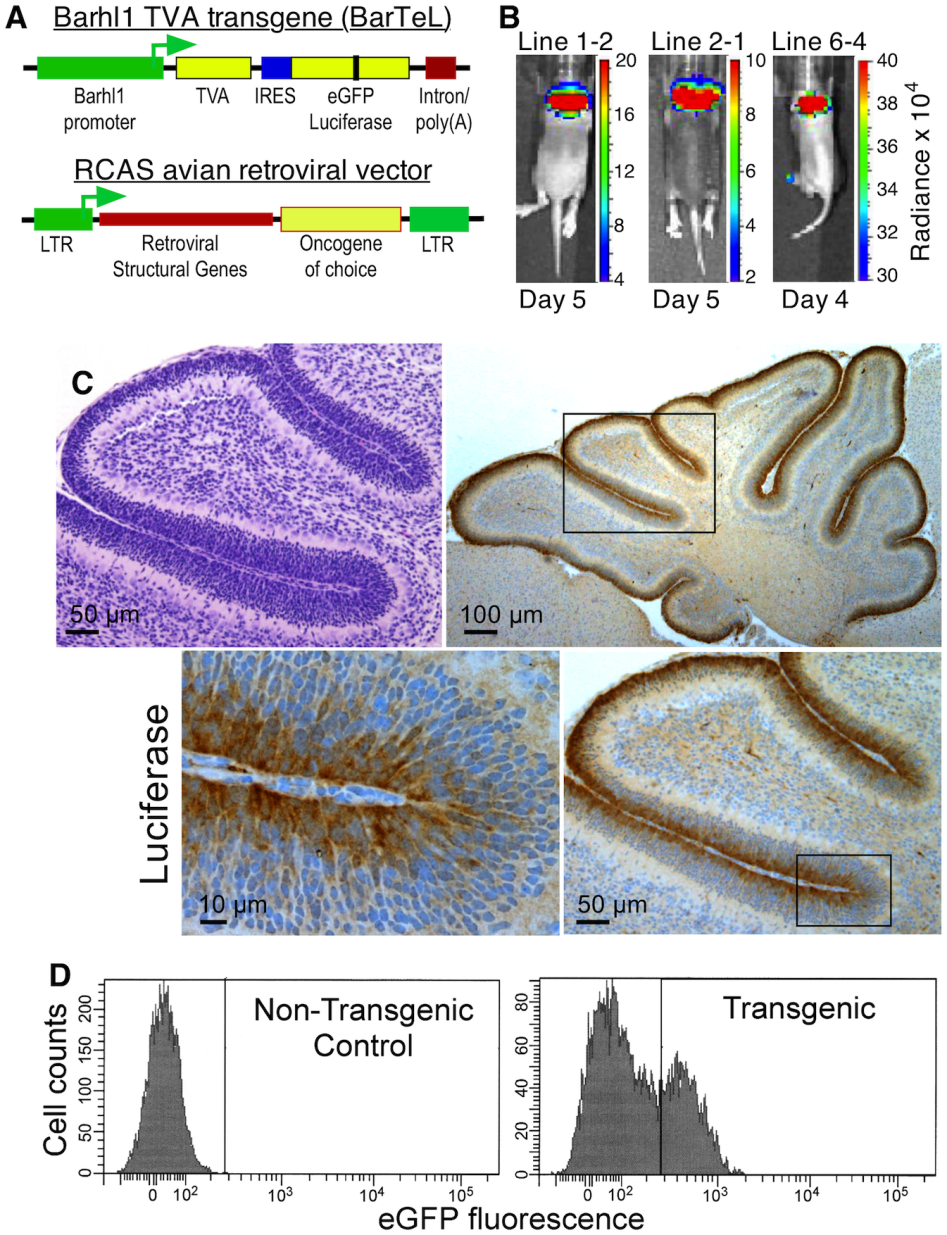
The BarTeL transgene is specifically expressed in EGL of neonatal mice. (A) Structure of the BarTeL transgene and RCAS retroviruses. In BarTeL, the mouse *Barhl1* promoter drives expression of a quail *Tva* cDNA. An IRES allows co-expression of an eGFP-luciferase fusion protein. The RCAS replication competent retroviral vectors express the inserted oncogenes via a splice acceptor located immediately upstream of the cloning site.(B) Bioluminescence imaging shows expression of the BarTeL transgene in the cerebellar region of three transgenic lines. Pups from lines 1–2, 2–1 and 6–4 were injected with luciferin and examined by optical imaging for bioluminescence. Age at imaging is indicated under the panels. (C) Histology and immunohistochemistry for luciferase transgene protein in brains of BarTeL mouse pups. H&E and successive magnifications (boxed, clockwise) of anti-luciferase staining of sagittal sections of transgenic mouse pup cerebella are shown. (D) eGFP expression in BarTeL cerebellar cells. Dissociated cerebella from BarTeL and nontransgenic mouse pups were analyzed by flow cytometry to demonstrate eGFP expression and the ability to sort eGFP-positive cells, indicative of BarTeL transgene expression.

We next built a transgene comprised primarily of the *Barhl1* promoter fragment, a *Tva* avian retrovirus receptor cDNA, an internal ribosome entry site (IRES), and an eGFP-luciferase fusion protein cDNA (Fig 2A). Using this transgene we produced three independent transgenic lines that exhibited strong restricted luciferase expression in the cerebellar region as revealed by bioluminescence imaging at four and five days of age (Fig 2B). We used line 1–2 for additional analysis in the present study. These transgenic mice are hereafter termed BarTeL mice (Barhl1, TVA, eGFP-Luciferase).

To further delineate the cells that express the transgene in the cerebella of neonatal BarTeL mice, we performed immunohistochemistry using antibodies against firefly luciferase. This showed that the transgene was expressed in the EGL as expected (Fig 2C). Examination at higher magnification showed that more intense staining was observed in the proliferative outer layer of the neonatal EGL than in the postmitotic inner layer (Fig 2C). Thus, the transgene is expressed in cGNP cells as designed.

To test the eGFP portion of the eGFP-luciferase fusion protein for function, we produced a single-cell suspension of cerebellar cells from three-day-old BarTeL mouse pups and analyzed them by flow cytometry. This analysis showed that an eGFP-positive peak of cells was detected and was separable from the eGFP-negative cell peak. Approximately 20–30% of cells were gated into the eGFP-positive window in multiple experiments (Fig 2D). The ability to specifically sort these eGFP-positive cGNP cells will be useful in studies where highly purified cGNPs are desired and in analyses of transgene expression-positive and expression–negative medulloblastoma cells.

**Fig 3:**
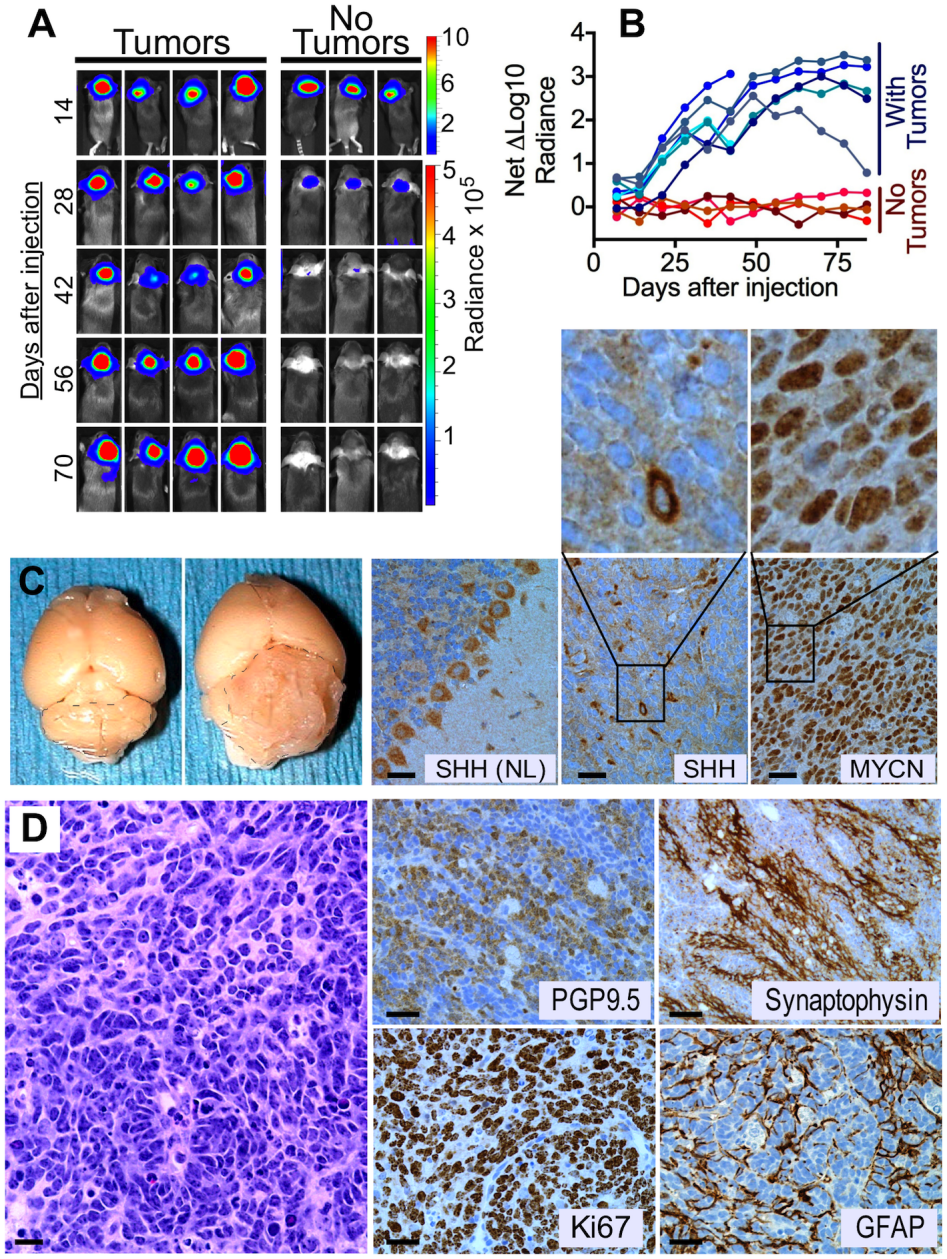
*ShhN* and *Mycn*^*T50A*,*S54A*^ cooperatively induce bioluminescent medulloblastomas *in vivo* in BarTeL mice. (A) BarTeL mice were injected with a mixture of chicken DF-1 cells producing RCAS-*ShhN* and RCAS-*Mycn*^*T50A*,*S54A*^ viruses and imaged for bioluminescence starting at 14 days post-injection. Shown are images at two week intervals of four mice that developed bioluminescent tumors (left) compared to three mice without tumor (right). (B) Time course of bioluminescent tumor formation in transgenic mice. A cohort of 11 transgenic mice, 10 of which were infected as in (A), were imaged weekly for 12 weeks. At each time point, net ΔLog10 flux values plotted for each mouse were determined by subtracting the mean of the log values of flux of non-tumor-bearing transgenic mice from the log of the flux of each mouse. A graph of unprocessed measurements before conversion to log values is provided separately (S2 Fig) to show the loss of bioluminescence by cGNPs during postnatal cerebellar development and the concomitant growth of tumor bioluminescence in those mice that developed medulloblastomas. (C) Medulloblastoma in whole BarTeL brain and immunohistochemistry for SHHN and MYCN. Gross appearance of a medulloblastoma in a formalin-fixed brain (second panel) compared to a normal brain (first panel) from a littermate. Staining with an anti-SHH antibody showed that only a minority of medulloblastoma cells produced SHHN protein (fourth panel). Purkinje cells in control normal (NL) adult cerebellum also stain positively with anti-SHH antibody (third panel). An anti-c-MYC antibody identified the MYC-tagged MYCN^T50A,S54A^ protein in most cells of the same tumor (fifth panel). The magnified panels above the SHH and MYCN panels show the hypercellularity of these tumors. Bar, 10 μm. (D) Histology and immunohistochemistry of a representative medulloblastoma induced by *ShhN* and *Mycn*^*T50A*,*S54A*^ viruses. Shown are an H&E-stained tumor section (left panel) and tumor sections stained with antibodies to PGP9.5 (ubiquitin carboxyl-terminal esterase L1), Synaptophysin, Ki67 and GFAP. Bar, 10 μm.

We show above that *Barhl1* expression in the cerebellum declines with age after the first weeks of life (Fig 1A). Thus, if the transgene's *Barhl1* promoter is functioning as expected, cerebellar expression of the transgene in postnatal mice should be limited to the early postnatal period and subside afterward. Indeed, bioluminescence imaging of young BarTeL mice showed that cerebellar transgene expression declines as expected and is reduced to undetectable levels by seven weeks of age (Fig 3A right panel, without tumors; S2 Fig), indicating that the *Barhl1* promoter fragment retains its expected temporal and spatial functional characteristics.

During bioluminescence imaging analyses we observed an incidental bioluminescent signal in the scrotal region of male mice beginning at 19 days of age. This timing suggested that the BarTeL transgene may be expressed in developing male germ cells, specifically the round spermatids, as these cells are known to appear in the developing mouse testis at 18–22 days of age [44, 45]. Immunohistochemical analysis of adult testis sections using anti-luciferase showed that strong expression was indeed present in round spermatids (S3 Fig).

### BarTeL mice produce bioluminescent medulloblastomas after infection with oncogene-containing RCAS vectors

To examine the utility of BarTeL mice in producing medulloblastomas modeling the SHH subgroup, we injected cerebella of BarTeL pups at 1–3 days of age with nontumorigenic chicken DF-1 cells producing either RCAS-*ShhN* or RCAS-*Mycn*^*T50A*,*S54A*^, or with DF-1 cells producing both viruses. MYCN amplification is known to be an important marker for poor prognosis in the SHH subgroup of human medulloblastomas [46–50], and the combination of *Shh* and *Mycn* have been shown to cause medulloblastomas in mice [51]. Targeted cerebellar expression of *Mycn* in transgenic mice was also shown to give rise to medulloblastomas with classic and large cell anaplastic pathologies [52]. We initially chose to use a T50A, S54A double mutant of Mycn because either mutant alone causes at least a 5-fold extension of protein half-life [53], which mimics the frequent overexpression and occasional amplification of *MYCN* in human medulloblastoma. Infection of BarTeL mice with either the RCAS-*ShhN* or RCAS-*Mycn*^*T50A*,*S54A*^ vector induced medulloblastomas in 20% (6 of 30) and 3% (1 of 35) of the mice, respectively. Mice injected with cells producing both viruses developed medulloblastomas in 47% (26 of 55) of mice, demonstrating the expected oncogenic cooperativity of these genes.

BarTeL mice express both the TVA receptor and the eGFP-luciferase fusion protein in cGNPs. This expression, as measured by luciferase-catalyzed bioluminescence, declined in parallel with the decrease of *Barhl1* RNA levels in the cerebella of maturing mice (Fig 3A right panel and Fig 1A, respectively). Given the *BARHL1* expression in human medulloblastomas (Fig 1B and S1 Table), we anticipated that developing mouse medulloblastomas would maintain the primitive phenotype of cGNPs, including expression of the *Barhl1* promoter-driven transgene, despite the increasing age of the mice. Indeed, medulloblastomas that developed in BarTeL mice showed bioluminescence that was readily detectable above the declining bioluminescence of the maturing non-tumor cerebellum beginning at three weeks of age (Fig 3A and 3B). Accordingly, the net bioluminescence increased as the tumors grew (Fig 3B). This early detection of the bioluminescent medulloblastomas enabled identification of mice with tumors many weeks before they developed neurological symptoms and required euthanasia.

At necropsy, we observed that all medulloblastomas developed on the surface of the cerebellum, consistent with their origin in the EGL of these mice (Fig 3C). Immunostaining for the Mycn^T50A,S54A^ protein in tumors infected with both viruses shows positive staining in a majority of tumor cells (Fig 3C). Antibody against the SHHN protein stained only a minority of cells, suggesting that the tumor likely consists of a mixture of cells infected by either RCAS-*ShhN* or RCAS-*Mycn*^*T50A*,*S54A*^. These findings, together with the ability of RCAS-*ShhN* alone to be tumorigenic, are consistent with the notion that SHHN acts in both autocrine and paracrine fashions in the production of medulloblastomas.

Histopathological and immunohistochemical analyses showed cellular tumors composed of primitive cells displaying a high nuclear/cytoplasmic ratio, focal nuclear molding and elevated mitotic activity (Ki67; Fig 3D). Neuronal differentiation was present and is highlighted by areas stained positively by the neuronal markers synaptophysin and PGP9.5 (Fig 3D). Thus, the overall morphological and immunohistochemical features of the tumors are generally characteristic of medulloblastomas.

These data demonstrate that the BarTeL mice function as designed: 1) they express the TVA and eGFP-luciferase proteins in the transient cGNP cells of the postnatal EGL, 2) they enable infection of these cells at early postnatal times with avian retroviral vectors, leading to medulloblastoma formation, and 3) they allow bioluminescence imaging of tumor formation, which should enable noninvasive monitoring of medulloblastoma development as well as therapy-induced regression in preclinical studies of new therapeutics.

### *Ex vivo*-infected BarTeL cerebellar cells efficiently cause tumors when transplanted

**Fig 4:**
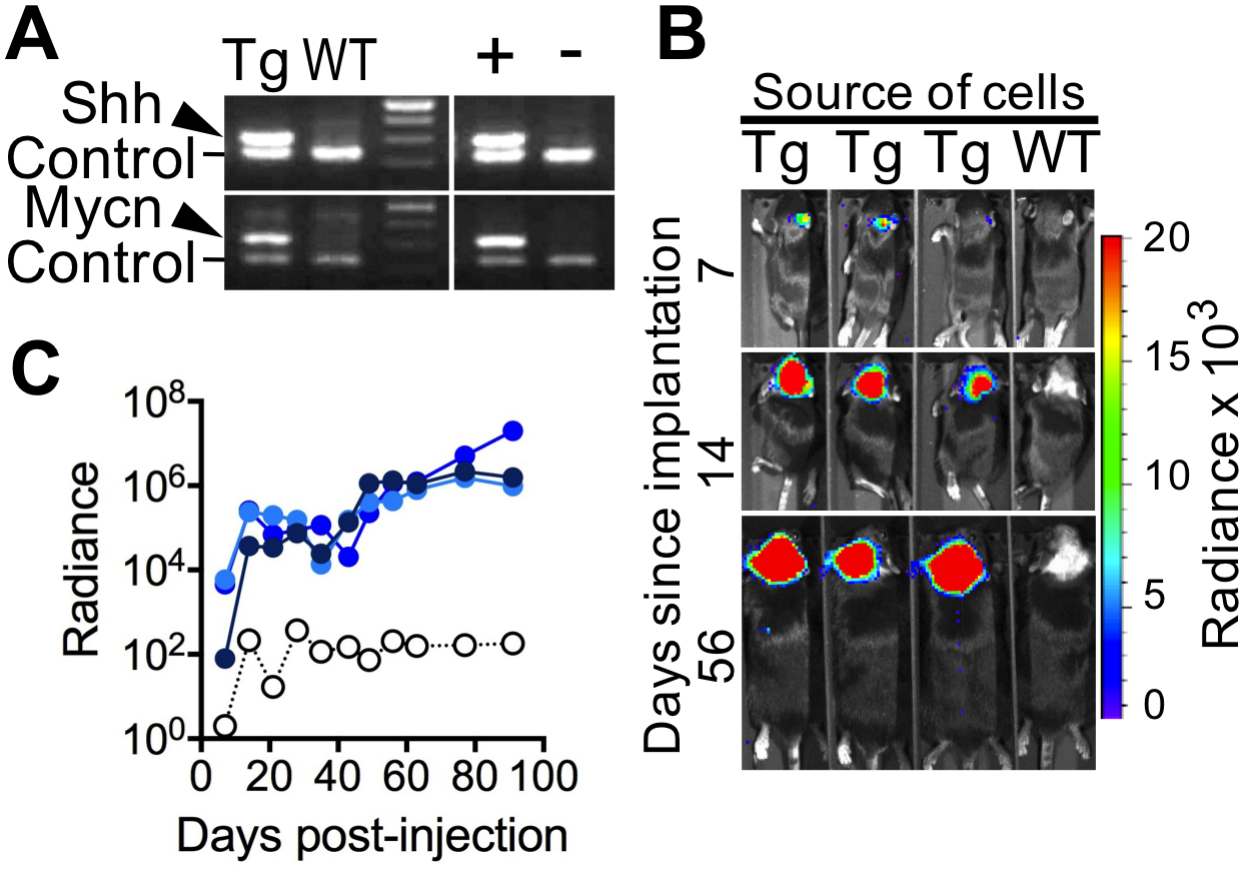
Neonatal BarTeL cGNP cells infected *ex vivo* with a mix of RCAS-*ShhN* and RCAS-*Mycn*^*T50A*,*S54A*^ viruses efficiently develop into bioluminescent medulloblastomas when orthotopically injected into nontransgenic cerebella. (A) PCR assay to detect the specific infection of transgenic cGNPs in culture. Cerebella from transgenic (Tg) and non-transgenic wild type (WT) mice were dissociated, put into culture, infected with RCAS-*ShhN* and RCAS-*Mycn*^*T50A*,*S54A*^ viruses and then used for preparation of genomic DNA as described in Materials and Methods. PCRs of these genomic DNAs were performed to test for presence of RCAS-*ShhN* (upper panels) or RCAS-*Mycn*^*T50A*,*S54A*^ (lower panels) proviral DNAs. Positive control template (+) was DNA from a *ShhN* and *Mycn*^*T50A*,*S54A*^-induced mouse medulloblastoma, and negative control template (–) was DNA from an uninfected transgenic cerebellum. Control primers that amplify an intron region of a single-copy gene (*Fgfr2*) were included in all PCRs. The third lane contains 100-bp markers. Thirty-five PCR cycles were used. PCR Primers are listed in S2 Table. (B) Bioluminescent tumors induced by transplanting *ex vivo* infected transgenic cerebellar cells into non-transgenic cerebella. Dissociated cerebellar cells from transgenic (Tg) or non-transgenic (WT) neonatal mice were exposed to RCAS-*ShhN* and RCAS-*Mycn*^*T50A*,*S54A*^ viruses in culture, harvested, injected into the cerebella of non-transgenic neonatal B6D2F1 mice and imaged at the times shown. (C) Time course of bioluminescent tumor formation in recipient mice orthotopically injected with *ex vivo*-infected transgenic cerebellar cells. Bioluminescence measurements of the mice shown in (B) were plotted over time. Solid lines (blue) denote mice receiving transgenic cerebellar cells; dotted line (black, empty circles) denotes mouse receiving non-transgenic cells.

The RCAS-TVA system, which restricts virus infection to only those mouse cells producing the TVA receptor, lends itself to *ex vivo* infection of TVA-positive cells placed in culture followed by transplantation of the cells into a syngeneic or immunodeficient host. We tested this *ex vivo* approach to tumor formation with the BarTeL mice. We first exposed freshly-dissociated primary cerebellar cells of both transgenic and nontransgenic pups to a mixture of RCAS-*ShhN* and RCAS-*Mycn*^*T50A*,*S54A*^ viruses in culture and tested them 24 hours later for evidence of infection (proviral DNAs) by PCR. Transgenic, but not nontransgenic, cerebellar cells were efficiently infected by both viruses in culture, suggesting that only *Tva*-expressing cGNP cells were infected in the transgenic cell culture (Fig 4A). We removed aliquots of these transgenic and nontransgenic cerebellar cells after 4 hours of exposure to viruses and implanted the cells into cerebella of nontransgenic B6D2F1 pups. We found that only transgenic, but not nontransgenic, virus-exposed cells were capable of inducing tumors in implanted mice, and the tumors were bioluminescent as would be predicted (Fig 4B). A time course of the growth of these tumors as measured by bioluminescence imaging (Fig 4C) showed that they grew with increased initial kinetics compared with the *in vivo* direct injection approach (Fig 3B), presumably due to an increase in the number of initially infected cells per mouse.

### A bicistronic RCAS vector increases tumor incidence and lethality

**Fig 5:**
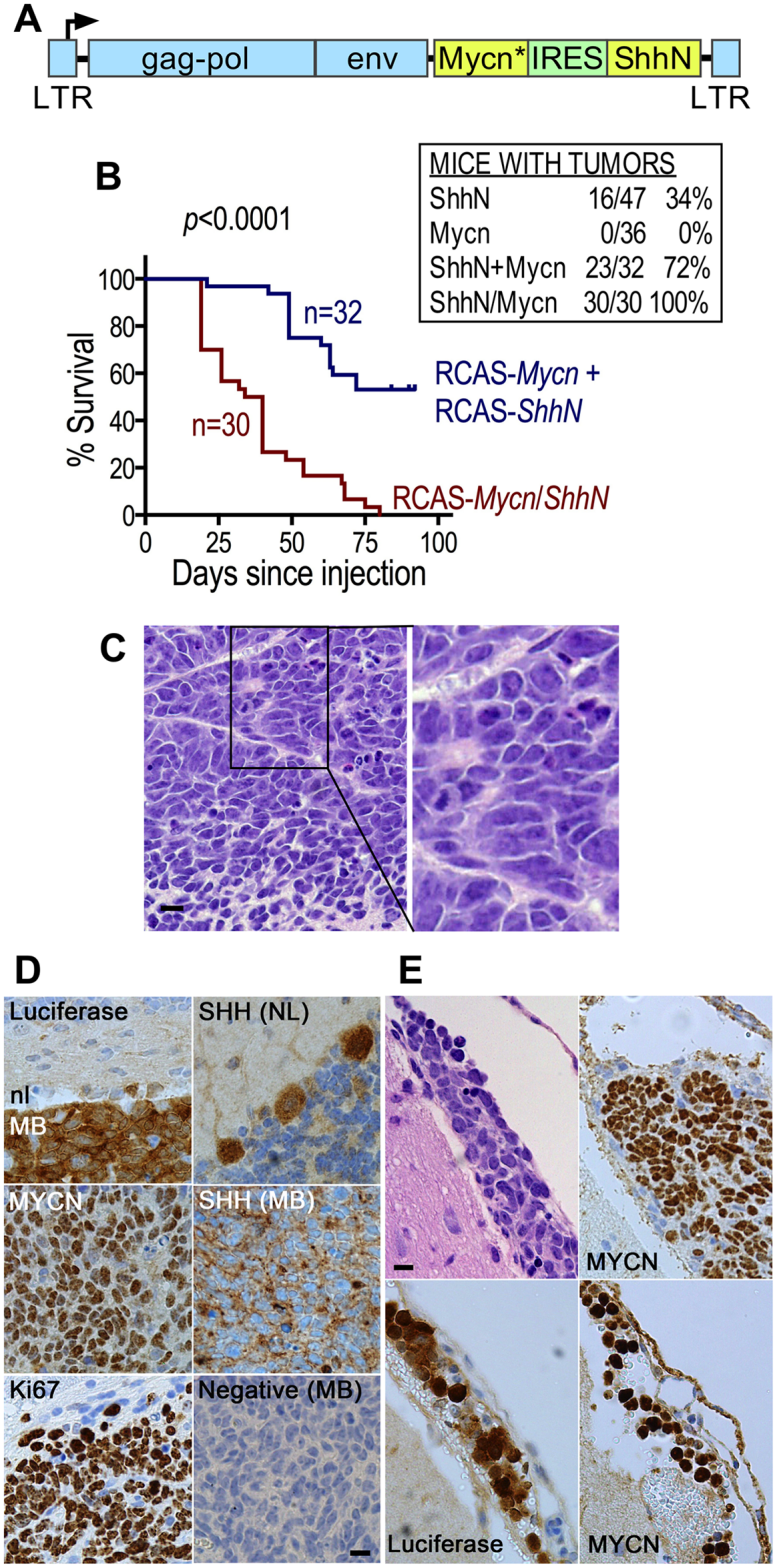
Bicistronic RCAS virus expressing both *Mycn*^*T58A*^ and *ShhN* increases tumor incidence and lethality. (A) Map of the bicistronic RCAS vector. Mycn* denotes *Mycn*^*T58A*^. Not to scale. (B) Kaplan-Meier curves of time to tumor-related euthanasia of mice infected with the RCASBP(A)ΔF1’-*Mycn*^*T58A*^*/ShhN* bicistronic virus (maroon) compared to a mixture of the RCASBP(A)ΔF1’-*Mycn*^*T58A*^ and RCASBP(A)ΔF1’-*ShhN* single-gene viruses (blue). The inset table shows the incidence of medulloblastomas in BarTeL mice infected with the bicistronic virus (ShhN/Mycn), the separate single-gene viruses and a mixture of the two single-gene viruses (ShhN + Mycn) derived from the bicistronic plasmid. Mice were euthanized upon development of tumor-related symptoms. (C) H&E stain of a BarTeL bicistronic medulloblastoma. Typical field from an H&E stain of a medulloblastoma arising in the cerebellum of a BarTeL mouse that was injected with the ShhN/Mycn-containing bicistronic virus. Right panel is a magnified view. Bar, 10 μm. (D) Immunohistochemical staining of a representative medulloblastoma (ShhN/Mycn bicistronic virus) with antibodies to the indicated proteins. Luciferase staining demonstrates the border between normal cerebellum (nl, negative) and the tumor (MB; positive). MYCN expression is detected by staining with antibody to the human MYC epitope tag and shows that almost all cells in the tumor express nuclear *Mycn*^*T58A*^. Ki67 staining shown is at the border of the tumor, with the upper left area being adjacent normal brain. SHH staining is shown in medulloblastoma (MB) compared to the normal (NL) cerebellum, the latter of which demonstrates SHH expression in three Purkinje cells at the border between the molecular layer (upper left in image) and the IGL. Negative control of medulloblastoma was stained with nonspecific IgG primary antibody. Bar, 10 μm. (E) Leptomeningeal dissemination and spread in brains from bicistronic virus-infected mice. Shown are an H&E stain (upper left; note the overlying meninges) and staining with antibodies to luciferase (lower left) and the MYC epitope tag of MYCN^T58A^ (lower right) of areas in the same leptomeningeal dissemination over the hemisphere. In a different mouse, the upper right panel demonstrates leptomeningeal spread over normal cerebellum, which is contiguous to the main tumor mass a few millimeters away, outside the right lower area of the panel. Bar, 10 μm.

As presented in Fig 3C, infection of cGNPs *in vivo* with a mixture of two RCAS vectors carrying different oncogenes produces tumors that appear not to be derived from doubly infected cells, as shown by the lack of SHHN staining in most tumor cells. To ensure introduction of both genes into each infected cGNP and further improve the tumor model, we constructed a bicistronic RCAS vector capable of expressing both *Mycn*^*T58A*^ and *ShhN* from a single integrated provirus *via* an IRES (Fig 5A). Injection of DF-1 cells producing this bicistronic virus showed that it was highly potent in inducing bioluminescent medulloblastomas: 100% of injected mice rapidly developed large tumors with 73% of mice requiring euthanasia by 40 days post-infection (Fig 5B and 5C). The resulting tumors displayed characteristics of large cell/anaplastic medulloblastoma, including large nuclei, numerous mitoses, nuclear molding and cell wrapping (Fig 5C). Ki-67 staining further indicates significant mitotic activity (Fig 5D). As expected, these tumors expressed both *ShhN* and *Mycn*^*T58A*^ in the majority of cells (Fig 5D), in contrast to the tumors induced by mixed single-gene vectors in Fig 3C, where *ShhN* was only expressed in a minority of cells. Infection of mice with the bicistronic virus produced a higher incidence of tumors and shortend survival versus mice infected with mixture of two single-gene viruses derived from this same bicistronic vector (Fig 5B). As controls, these single-gene viruses, which expressed either *ShhN* or *Mycn*^*T58A*^, produced tumors only at incidences of 36% and 0%, respectively (Fig 5B). Taken together, these data demonstrate that introduction of both oncogenes into infected cells via a single bicistronic virus significantly increases the frequency and accelerates the lethality of medulloblastomas in this model. Interestingly, immunohistochemical examination of two sections from each of six tumor-bearing mouse brains from bicistronic vector-infected mice revealed an apparent luciferase-expressing leptomeningeal dissemination on the hemispheric surface in one of the mice and spread of a primary medulloblastoma into the leptomeninges in a second mouse (Fig 5E). Analyses of additional tumors will be required to determine if leptomeningeal disseminations are a common characteristic of tumors produced by the bicistronic virus.

### Molecular subgrouping of BarTeL *ShhN/Mycn*^*T58A*^ medulloblastomas

**Fig 6:**
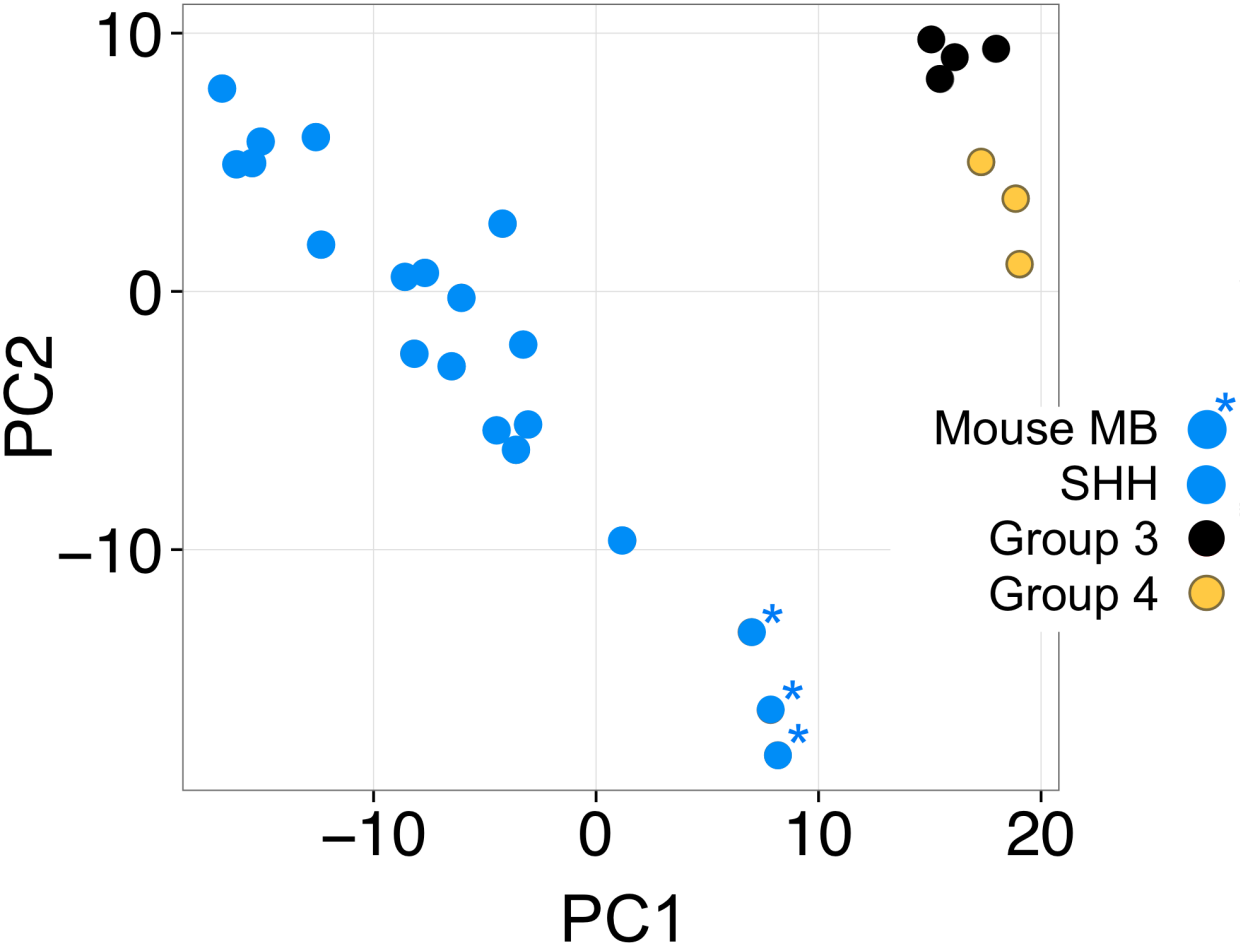
Molecular subgrouping analysis of BarTeL *ShhN/Mycn*^*T58A*^ medulloblastomas. Scatter plot of the first two principle components (PC) of human medulloblastoma samples (GSE4036) and BarTeL *Shh/Mycn*^*T58A*^ (bicistronic virus-induced) medulloblastomas. Principle component analysis used a subset of highly variable genes (n = 631) among human medulloblastoma. The expression data from human genes and their mouse orthologs were normalized against the corresponding species-specific normal cerebella.

A bioinformatics strategy was developed to compare mouse medulloblastomas to human medulloblastoma groupings. This comparison was performed with the caveat that we induced tumors with only two of the possible driver genes of SHH tumors and without any of the large chromosomal abnormalities common to this subgroup. This strategy normalized gene expression data by using normal cerebellum data from each species. Consistent with the use of *Shh* and *Mycn* genes to create the mouse tumors, a clustering analysis of highly variable genes among human medulloblastoma subgroups demonstrated that the distinct cluster of BarTeL medulloblastoma samples was closest to that of human SHH subgroup medulloblastomas. The Group 3 and Group 4 tumors, as expected, clustered together and were well separated from the other groups (Fig 6).

## Discussion

To study the cooperative effects of oncogene and tumor suppressor gene combinations in typical genetically engineered mouse models for cancer, including transgenic and knockout models, labor and time intensive production of each line and/or crossbreeding are generally required. These models often also lack the ability to mimic the somatic acquisition of mutations observed in human tumors and require further crossbreeding to equip them with facile tumor cell sorting and *in vivo* tumor imaging capabilities. In this study, we have surmounted all of these obstacles with the design of a new model for medulloblastoma, the BarTeL mouse. This model takes advantage of a promoter fragment that we isolated from the *Barhl1* gene, which provides strong transient expression of the TVA-eGFP/luciferase transgene in GNP cells of the developing cerebellum.

This BarTeL mouse has a number of advantages and potential uses. 1) The RCAS-TVA system enables evaluation of cooperation of multiple oncogenes in a single mouse without the need for crossbreeding. 2) The delivery of oncogenes to limited somatic cGNP cells reproduces the circumstances in human medulloblastomas whereby tumors arise in only a subset of cells within an otherwise normal cellular environment. 3) Bioluminescence imaging facilitates early medulloblastoma detection before mice become symptomatic. 4) The easily detectable early bioluminescence of the predictable medulloblastomas lends itself to studies evaluating the efficacy of therapeutics. 5) Using BarTeL, it is possible to achieve 100% tumor incidence with possible leptomeningeal metastasis when using collaborating oncogenes in the bicistronic RCAS vector. 6) *Barhl1* promoter-driven eGFP-expressing medulloblastoma cells are likely to retain undifferentiated properties compared to more differentiated tumor cells that have lost expression from the *Barhl1* promoter thus potentially enabling effective isolation of putative tumor initiating cells by FACS. 7) eGFP also allows purification of native neonatal cGNPs. These cells can be used for experiments such as *ex vivo* transduction by RCAS viruses for orthotopic implantation in normal mice or, if testing the effect of the microenvironment, implantation in transgenic or knockout mice. 8) The early-stage temporally and spatially restricted expression of *Barhl1* promoter-driven eGFP-luciferase will facilitate studies of cerebellar and cGNP/EGL development. Thus, our new BarTeL mouse model provides an array of opportunities for investigations in brain development and medulloblastoma biology and therapeutics.

The transient expression of the *Barhl1* promoter predicted that the BarTeL transgene’s eGFP-luciferase fusion protein and its associated bioluminescence would decline as non-tumor-bearing mice matured. Indeed, postnatal luciferase expression in the cGNPs of newborn transgenic mice remained at high levels for only a few weeks after birth and decreased afterward as expected. In contrast, as we hypothesized, luciferase expression and the associated bioluminescence in the medulloblastomas that arose from these cGNPs was retained and increased as the tumors grew. Thus, the developing tumors could be monitored by bioluminescence imaging over time with insignificant or no interfering background. In most cases, with the possible exception of the most rapidly growing bicistronic vector-induced tumors, we were able to detect these bioluminescent tumors weeks before the mice became symptomatic. If early detection is desired when using the bicistronic virus, we have found that limiting the amount of virus or virus-infected DF-1 cells injected into neonatal BarTeL mice will allow detection of most or all tumors before the onset of symptoms.

The new bicistronic RCAS vector expressing *Mycn*^*T58A*^ and *ShhN* was very efficient at medulloblastoma induction, resulting in tumors in 100% of infected mice. This vector overcomes the inefficiency of infecting single cells via a mixed infection with two single-gene vectors. Future use of bicistronic RCAS vectors in *Tva* models, within packaging limitations, may allow detection of oncogene cooperation and generation of new *Tva* models that may otherwise not be possible or practical with single-gene vectors. We note that a mixed infection with single-gene vectors that were derived from the bicistronic vector produced a higher incidence of tumors compared with our first generation of single-gene vectors. This is likely due to one or more changes made during the construction of the bicistronic virus and possibly also to a change in virus production. For example, the bicistronic virus and its respective single-gene derivatives included the following: the longer isoform of MYCN mutated at T58A instead of the shorter isoform mutated at both T50A and S54A, an improved Kozak sequence for *ShhN*, presence of an IRES in the *ShhN* single-gene derivative, RCASBP(A)ΔF1’ instead of RCASBP(A), and use of freshly transfected DF-1 cells for virus production.

In this study, we used *Mycn* mutants designed to produce a stabilized protein to help mimic the *MYCN* amplification seen in some SHH subgroup medulloblastomas. Although we have not tested wild type *Mycn* here, very early studies in ongoing work using a different vector suggest that it is similarly capable of producing medulloblastomas in BarTeL mice as mutant *Mycn* is, in combination with Shh.

*Barhl1* is a homeobox-containing transcription factor gene and an ortholog of the *BarH* genes of Drosophila, which are required for the morphogenesis and fate determination of fly external sensory organs [54]. In vertebrates, *Barhl1* is essential for the migration of cerebellar granule cells and the maintenance and consequent survival of precerebellar neurons, cerebellar granule cells, cochlear hair cells of the inner ear, and the zonal layer neurons in the superior colliculus [37, 55, 56]. Target gene regulation can occur either by activation or repression of transcription. *Barhl1* is known to be regulated by ATOH1 and by itself and is predicted to be a major target of the *Lmx1a* master regulator gene, as described further below [57–59].

We chose the *Barhl1* promoter for our mouse model based on its specific expression in human medulloblastomas by SAGE analysis as well as its spatial and temporal expression pattern in neonatal mouse EGL. In other parts of the neonatal brain, a lower level of *Barhl1* gene activity has been detected in specific locations including the pontine nuclei, mammary nuclei of the hypothalamus, the inferior and superior colliculi and in cochlear hair cells of the inner ear [37, 55]. Although beyond the scope of the present study, future comprehensive immunohistochemical or in situ hybridization analysis of BarTeL brains may identify transgene expression in these non-cerebellar locations, which may allow BarTeL to model additional tumors and neurological diseases.

Several lines of evidence from our study suggest that medulloblastomas arising in BarTeL mice with the oncogenes used here represent the SHH subgroup of medulloblastomas. First, we demonstrate that the BarTeL transgene is expressed within the proliferating outer cGNP layer of the EGL. cGNPs are known cells of origin for SHH subgroup medulloblastomas [8–11]. Second, tumor induction was dependent upon infection with viruses producing SHHN. Third, the clustering pattern of BarTeL medulloblastomas was closest to that of human SHH medulloblastomas

Recently, the *BARHL1/DDX31* super-enhancer was identified as an element that activates the *GFI1B* oncogene when fused to it by chromosomal deletion in subsets of Group 3 and Group 4 medulloblastomas [60]. Super-enhancers generally function in a cell-type specific manner and often regulate key cell identity genes, primarily encoding transcription factors, which are required for tumor cell identity and maintenance [61–64]. As *BARHL1* can be controlled by this super-enhancer [58, 60], it seems reasonable to suspect that the BARHL1 homeobox protein may participate in specifying identity for the cells of origin for Group 3 and Group 4 medulloblastomas. This notion is supported by the high level of expression of *BARHL1* in Group 3 and Group 4 medulloblastomas (Fig 1C and S4 Fig). Moreover, a recent extensive analysis of medulloblastoma enhancers and super-enhancers showed that BARHL1 is the most highly expressed transcription factor in Group 4 medulloblastomas and also has one of the highest number of inferred target genes [58]. The same study also identified *BARHL1* as a predicted major target of the LMX1A master regulator Group 4 transcription factor. Additionally, the authors showed that the expression pattern of *Lmx1a* and two other Group 4 master regulators, *Eomes* and *Lhx2*, overlaps in the nuclear transitory zone of the cerebellar upper rhombic lip at embryonic day 13.5 (E13.5), which, with other phenotypic and molecular data, implicates these cells and their derivative precursors as putative cells of origin for Group 4 [58]. We find that *Barhl1* expression also overlaps with these master regulators in the nuclear transitory zone at E13.5 (S5 Fig). Together, these findings tempt us to speculate that BarTeL mice may provide a platform for modeling Group 3 and Group 4 medulloblastomas in addition to the SHH subgroup, if the cells of origin for these subgroups are infected with vectors carrying their group-specific driver genes at an appropriate time in cerebellar development. Studies to test these possibilities are in progress.

In the cerebella of our BarTeL mouse, the transgene is primarily expressed in the EGL and to a lesser extent in the postmitotic IGL. The closest mouse model to BarTeL is the nestin-*Tva* mouse, which, due to the widespread expression of nestin in stem-like cells throughout the central nervous system, has been very useful in inducing glioblastomas, medulloblastomas and brain stem gliomas [65–67]. A number of non-somatic, oncogene-specific and non-bioluminescent genetically engineered models for medulloblastomas have also been produced (reviewed by Markant and Wechsler-Reya [68]). The BarTeL model presented here distinguishes itself with its combination of somatically acquired oncogenes, cGNP targetability, variable and multiple oncogene versatility, *ex vivo* experimental possibilities, eGFP sortability, bioluminescence imaging capability and the yet theoretical potential to model multiple subgroups of human medulloblastomas. Thus, BarTeL mice provide a versatile model with opportunities for use in medulloblastoma biology and therapeutics.

## Supporting Information

S1 Fig Confirmation of PCR-amplified *Tva* gene function. Dishes of HEK-293T cells were either transiently transfected with an expression plasmid carrying the PCR-amplified *Tva* cDNA (+TVA Receptor) or were left untransfected (None). Two days later, one dish from each group of cells was exposed to a 10^−1^ dilution of unconcentrated RCASBP(A)-AP virus (10^−1^); the remaining two dishes were untreated (No Virus). After an additional two days, exposure of all four dishes to substrates of alkaline phosphatase to detect infected cells showed that only *Tva*-transfected cells were infectable by the RCASBP(A)-AP viral vector.
(TIFF)

S2 Fig measurements of cranial bioluminescence from BarTeL mice infected with avian retroviral vectors. Graphed are the raw data used to produce the graph in Fig 3B.
(TIFF)

S3 Fig Immunohistochemical staining of adult BarTeL testis with anti-luciferase shows transgene expression in round spermatids. (TIFF)

S4 Fig Expression of *BARHL1* is highest in the two worst-prognosis subgroups (Group 3, Group 4) of human medulloblastoma as well as the SHH subgroup. *BARHL1* gene expression data were obtained from the R2 genomic analysis and visualization platform (http://r2.amc.nl) and plotted according to subgroup. The database used was “Tumor Medulloblastoma MAGIC–Northcott– 285 –rma_sketch–hugene11t”.
(TIFF)

S5 Fig Overlapping expression of *Barhl1* with master regulator Group 4 transcription factor genes in putative cells of origin for Group 4 medulloblastomas. In situ hybridization images from the Allen Brain Atlas (http://developingmouse.brain-map.org) are shown for the Group 4 master regulators *Lmx1a*, *Eomes* and *Lhx2* and for the *Atoh1* and *Barhl1* transcription factor genes. The images for *Lmx1a*, *Eomes*, *Lhx2* and *Atoh1* are as presented by Lin et al. [58]. *Lmx1a*, *Eomes*, *Lhx2* are expressed in the nuclear transitory zone (downward arrowheads), which has been implicated to contain cells of origin for Group 4 medulloblastomas. *Atoh1* is a transcription factor expressed in a more rostral segment of the cerebellar rhombic lip (upward arrowheads) and often used as a marker expressed in neonatal EGL cells and SHH subgroup tumors. We show that *Barhl1* exhibits overlapping expression with the Group 4 master regulators in putative Group 4 cells of origin and with *Atoh1*.
(TIFF)

S1 Table SAGE identifies *BARHL1* as a gene expressed highly and frequently in human medulloblastomas. (DOC)

S2 Table Primer list for RT-PCRs in Figs 1 and 4. (DOCX)
