# Supplementary figures and images for: BarTeL, a Genetically Versatile, Bioluminescent and Granule Neuron Precursor-Targeted Mouse Model for Medulloblastoma
Source: PLoS One. 2016 Jun 16;11(6):e0156907. doi: 10.1371/journal.pone.0156907 (PMC4911170; doi:10.1371/journal.pone.0156907)

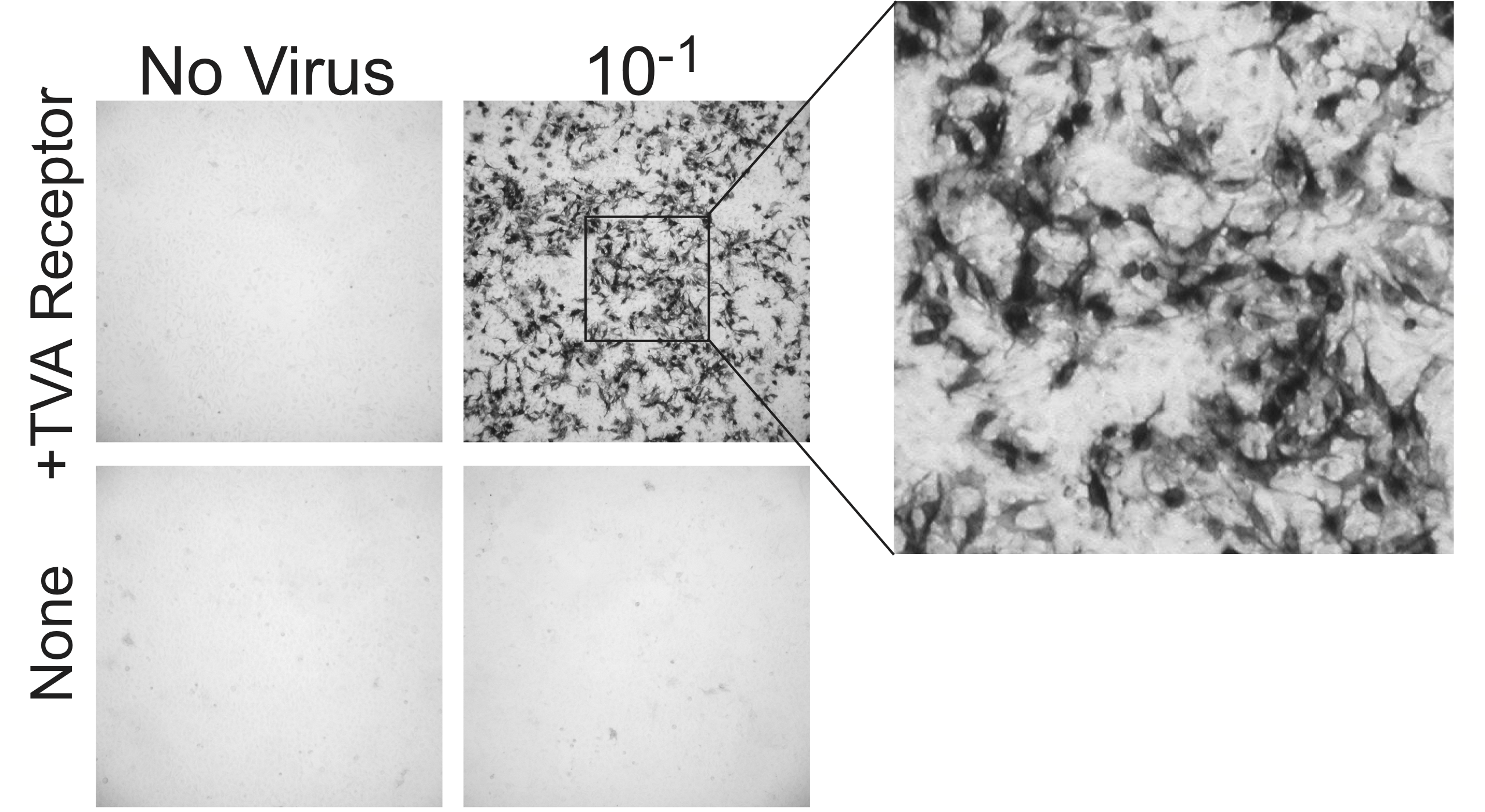

Supplement: S1 Fig — Dishes of HEK-293T cells were either transiently transfected with an expression plasmid carrying the PCR-amplified Tva cDNA (+TVA Receptor) or were left untransfected (None). Two days later, one dish from each group of cells was exposed to a 10−1 dilution of unconcentrated RCASBP(A)-AP virus (10−1); the remaining two dishes were untreated (No Virus). After an additional two days, exposure of all four dishes to substrates of alkaline phosphatase to detect infected cells showed that only Tva-transfected cells were infectable by the RCASBP(A)-AP viral vector. (TIFF) [file pone.0156907.s001.tiff]

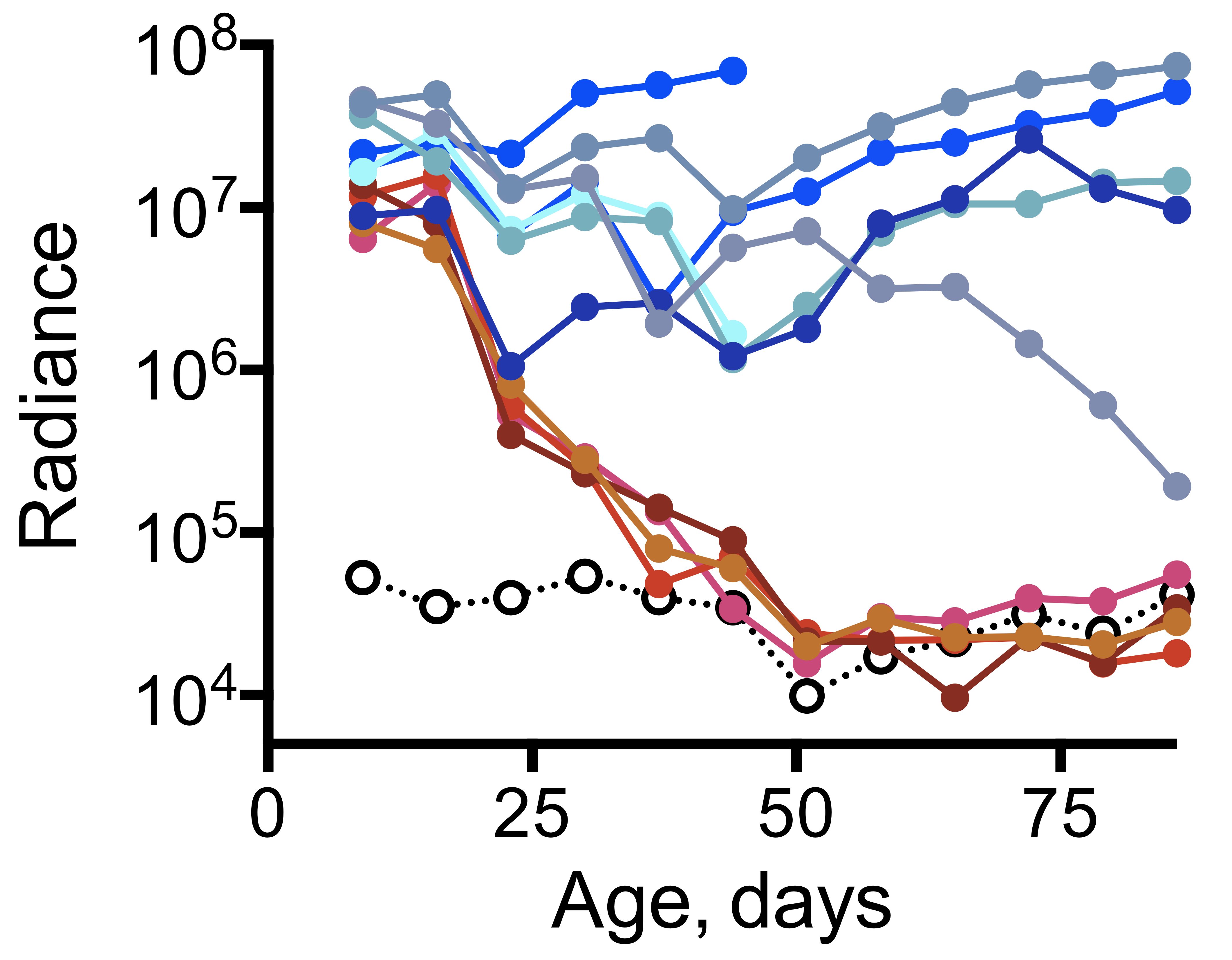

Supplement: S2 Fig — Graphed are the raw data used to produce the graph in Fig 3B. (TIFF) [file pone.0156907.s002.tiff]

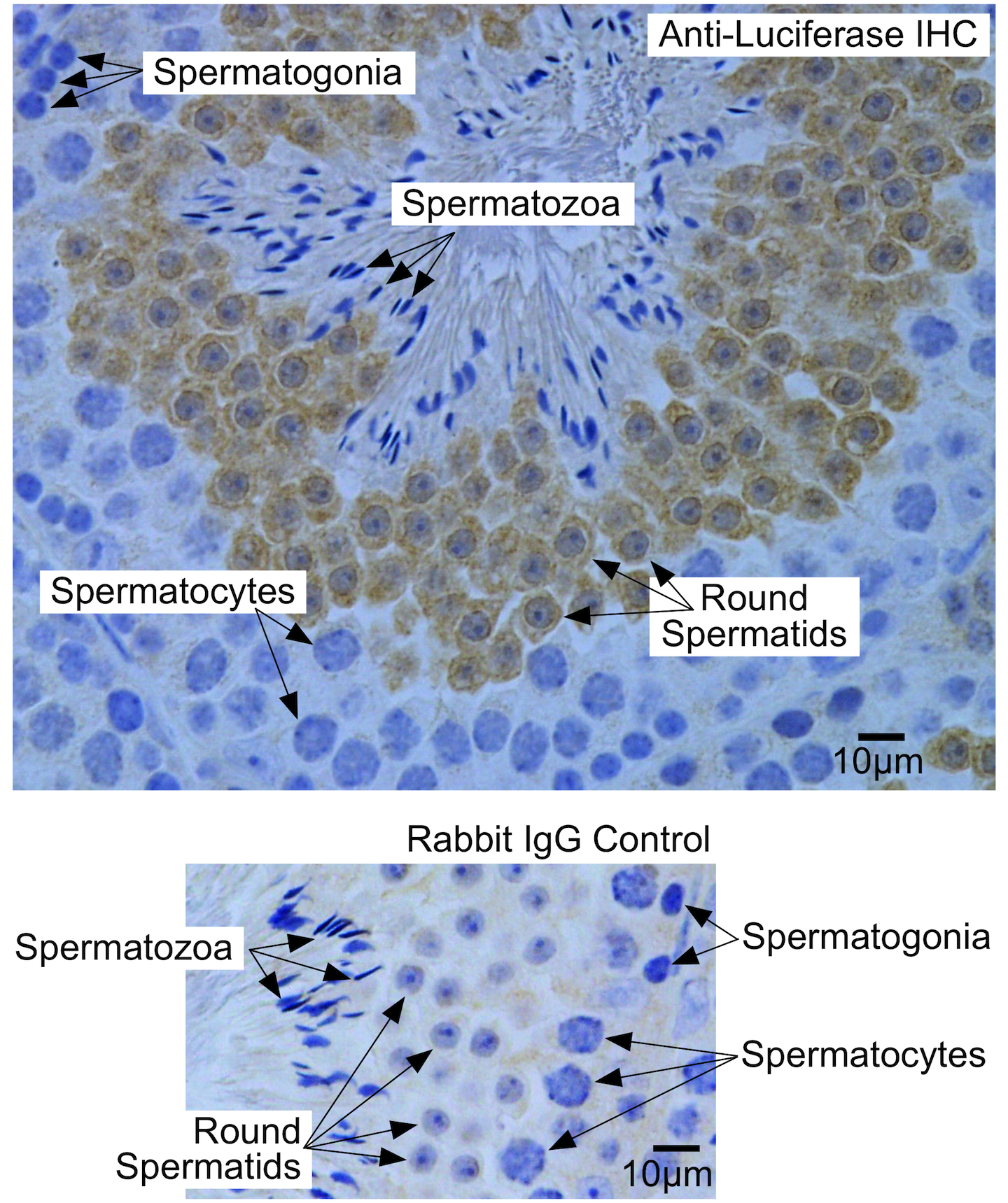

Supplement: S3 Fig — (TIFF) [file pone.0156907.s003.tiff]

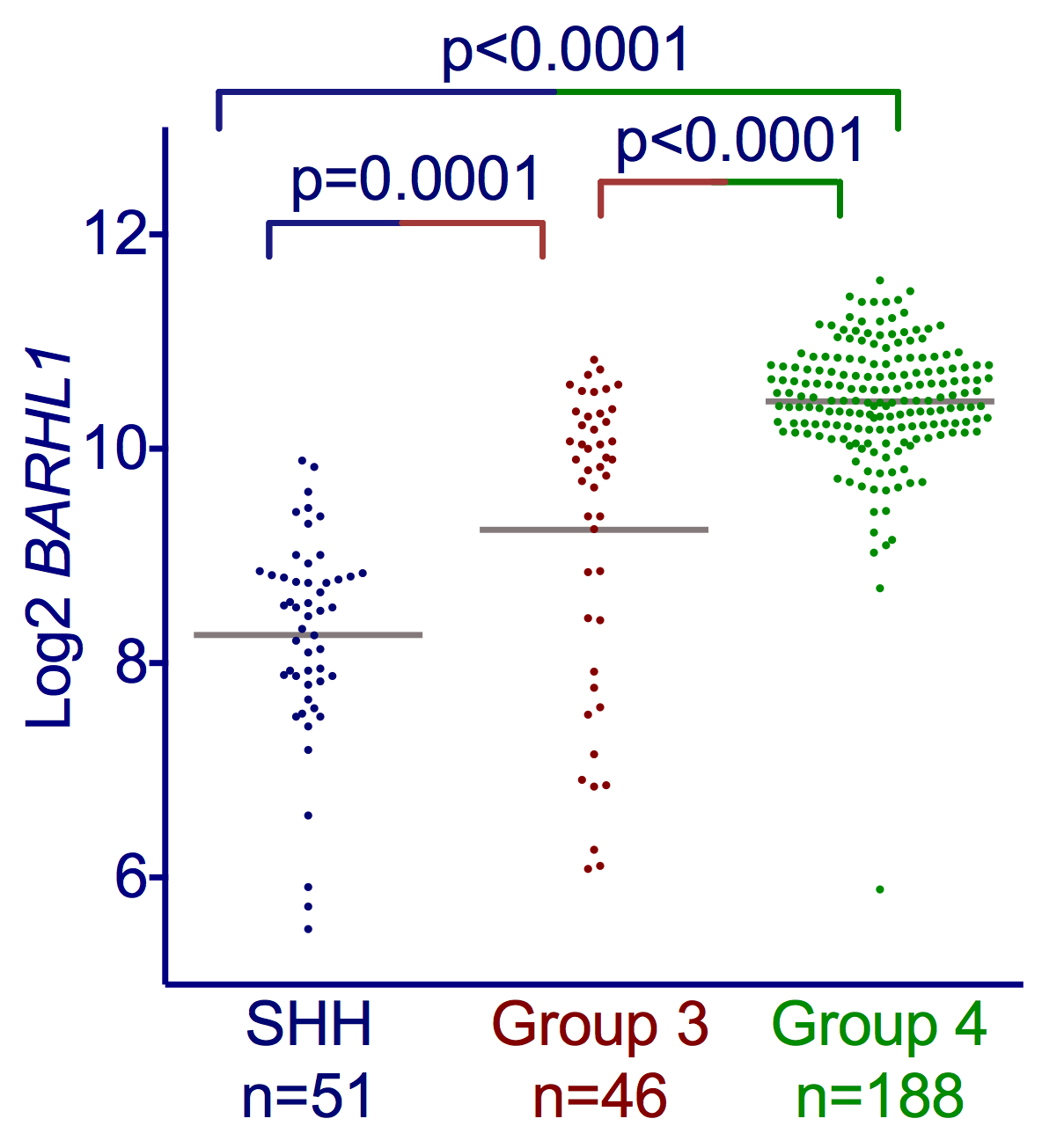

Supplement: S4 Fig — BARHL1 gene expression data were obtained from the R2 genomic analysis and visualization platform (http://r2.amc.nl) and plotted according to subgroup. The database used was “Tumor Medulloblastoma MAGIC–Northcott– 285 –rma_sketch–hugene11t”. (TIFF) [file pone.0156907.s004.tiff]

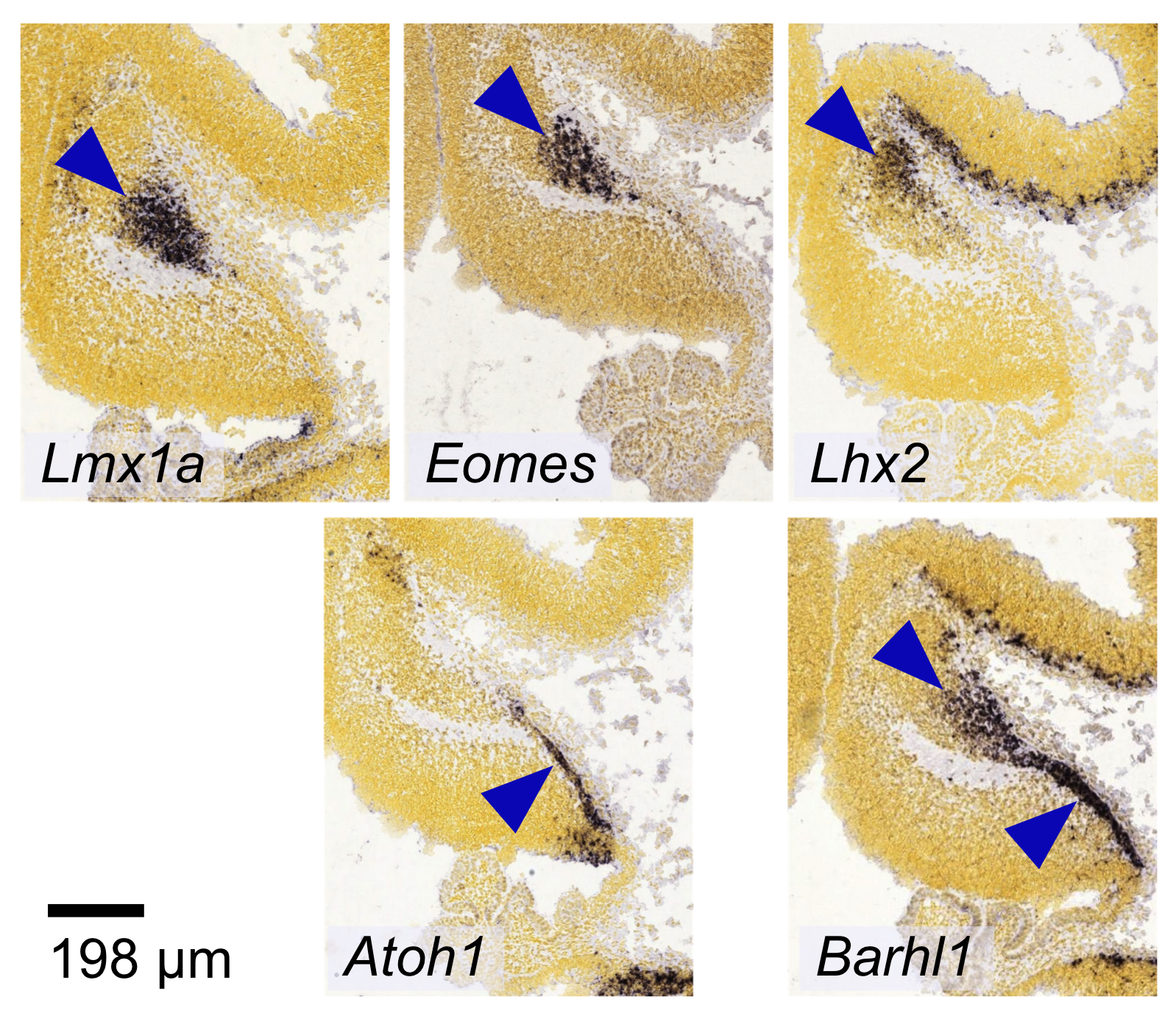

Supplement: S5 Fig — In situ hybridization images from the Allen Brain Atlas (http://developingmouse.brain-map.org) are shown for the Group 4 master regulators Lmx1a, Eomes and Lhx2 and for the Atoh1 and Barhl1 transcription factor genes. The images for Lmx1a, Eomes, Lhx2 and Atoh1 are as presented by Lin et al. [58]. Lmx1a, Eomes, Lhx2 are expressed in the nuclear transitory zone (downward arrowheads), which has been implicated to contain cells of origin for Group 4 medulloblastomas. Atoh1 is a transcription factor expressed in a more rostral segment of the cerebellar rhombic lip (upward arrowheads) and often used as a marker expressed in neonatal EGL cells and SHH subgroup tumors. We show that Barhl1 exhibits overlapping expression with the Group 4 master regulators in putative Group 4 cells of origin and with Atoh1. (TIFF) [file pone.0156907.s005.tiff]
