# Supplementary material for: BarTeL, a Genetically Versatile, Bioluminescent and Granule Neuron Precursor-Targeted Mouse Model for Medulloblastoma
Source: PLoS One. 2016 Jun 16;11(6):e0156907. doi: 10.1371/journal.pone.0156907 (PMC4911170; doi:10.1371/journal.pone.0156907)
Supplement: S1 Table — (DOC) [file pone.0156907.s006.doc]

**S1 Table. SAGE identifies *BARHL1* as a gene expressed highly and frequently in human medulloblastomas**

| **Tag** | **Symbol/ Accession** | **(Frequency) Libraries** | | **(Expression) Tags** | | **Tag Odds A:B***** | **P** |
| --- | --- | --- | --- | --- | --- | --- | --- |
|  |  | **A*** | **B**** | **A*** | **B**** |  |  |
| ***Top-ranked genes that have 1 or more Pool B tags.*** | | | | | | | |
| **AGCCCGTGAC** | **BARHL1** | 14 | 1 | 294 | 1 | 2678 | 0 |
| GATTGACTTA | unidentified | 13 | 1 | 76 | 1 | 692 | 0 |
| TATCTATGAA | Hs.399852 | 6 | 1 | 35 | 1 | 319 | 0 |
| AGATGGGTGC | CTNNB1 | 8 | 2 | 67 | 2 | 305 | 0 |
| CTTAAAGCTC | unidentified | 9 | 1 | 21 | 1 | 191 | 0 |
| TATATTCATC | WNT16 | 2 | 1 | 18 | 1 | 164 | 0 |
| CCAACCTAAT | Hs.453788 | 8 | 2 | 35 | 2 | 159 | 0 |
| GAGGCACCGT | EPHA8 | 12 | 3 | 50 | 3 | 152 | 0 |
| GGTCCTTTTT | TNIK | 12 | 3 | 48 | 3 | 146 | 0 |
| CAGTGTGGTC | TAF3 | 5 | 2 | 31 | 2 | 141 | 0 |
|  | | | | | | | |
| ***Top-ranked genes that have no Pool B tags.*** | | | | | | | |
| GACTGTAATC | unidentified | 7 | 0 | 22 | 0 | NaN | 0 |
| TGAACCATTA | BE300221 | 9 | 0 | 13 | 0 | NaN | 0 |
| TCCATTCTTA | INSM2 | 7 | 0 | 13 | 0 | NaN | 0 |
| GAGTGAGTCA | BI001417 | 2 | 0 | 12 | 0 | NaN | 0 |
| TGGCCTTGCA | unidentified | 3 | 0 | 12 | 0 | NaN | 0 |
| CTCGCAGCGG | AW821238 | 3 | 0 | 12 | 0 | NaN | 0 |
| TCTCCCCTAA | BF375312 | 9 | 0 | 12 | 0 | NaN | 0 |
| TCTGGTTCCA | Hs.538028 | 4 | 0 | 9 | 0 | NaN | 0 |
| CAAGTTATAT | AW006067 | 4 | 0 | 9 | 0 | NaN | 0 |
| CTGCAATTAT | R33612 | 3 | 0 | 9 | 0 | NaN | 0 |

This Serial Analysis of Gene Expression (SAGE) output was produced by the human SAGE Digital Gene Expression Displayer tool at the SAGE website (http://cgap.nci.nih.gov/SAGE). We used this tool to query SAGE data for a list of genes or accession numbers that are expressed primarily in human medulloblastomas (Pool A) versus human non-cerebellar cancers and normal tissues (Pool B), excluding cell lines. The top-ranked gene overall was BARHL1.

*Library Pool A = Medulloblastomas; n = 20

**Library Pool B = Non-cerebellar tumors and tissues; n = 166

***Tag Odds A:B ratio is the percentage of Pool A tags that are BARHL1 divided by the percentage of Pool B tags that are BARHL1.

NaN: not a number
