# Supplementary material for: BarTeL, a Genetically Versatile, Bioluminescent and Granule Neuron Precursor-Targeted Mouse Model for Medulloblastoma
Source: PLoS One. 2016 Jun 16;11(6):e0156907. doi: 10.1371/journal.pone.0156907 (PMC4911170; doi:10.1371/journal.pone.0156907)
Supplement: S2 Table — (DOCX) [file pone.0156907.s007.docx]

| **S2 Table. Primer list for RT-PCRs in Figures 1 and 4.** | | | | |  |  |  |
| --- | --- | --- | --- | --- | --- | --- | --- |
|  |  |  |  |  |  |  |  |
| For Fig. 1A |  |  |  |  |  |  |  |
|  |  |  |  |  |  |  |  |
| Barhl1 ex1 F | CCGGAAGAGACTGTCTGGAG | | |  |  |  |  |
| Barhl1 ex2 R | ACTGTCCCGAGAGCTGGAG | | |  |  |  |  |
|  |  |  |  |  |  |  |  |
| Gapdh mouse F | TGCGACTTCAACAGCAACTC | | |  |  |  |  |
| Gapdh mouse R | GGTCTGGGATGGAAATTGTG | | |  |  |  |  |
|  |  |  |  |  |  |  |  |
| For Fig. 1B |  |  |  |  |  |  |  |
|  |  |  |  |  |  |  |  |
| BARHL1 Hum F1 | GAAGGGACTGTTTGGAGACG | | |  |  |  |  |
| BARHL1 Hum R1 | GGACTGTCCCTGGAGCTG | | |  |  |  |  |
|  |  |  |  |  |  |  |  |
| NESTIN Human F1 | GTGGGAAGATACGGTGGAGA | | |  |  |  |  |
| NESTIN Human R1 | ACCTGTTGTGATTGCCCTTC | | |  |  |  |  |
|  |  |  |  |  |  |  |  |
| ATOH1/MATH1-Hu-F2 | CAACGACAAGAAGCTGTCCA | | |  |  |  |  |
| ATOH1/MATH1-Hu-R2 | CCTCGAAAGTCGAGAAGTGC | | |  |  |  |  |
|  |  |  |  |  |  |  |  |
| GAPDH-Hu-F | CAATGACCCCTTCATTGACC | | |  |  |  |  |
| GAPDH-Hu-R | TTGATTTTGGAGGGATCTCG | | |  |  |  |  |
|  |  |  |  |  |  |  |  |
| For Fig. 4A |  |  |  |  |  |  |  |
|  |  |  |  |  |  |  |  |
| RCASBPA F | CTGAGCTGACTCTGCTGGTG | | |  |  |  |  |
| Shh-N Orient R | GATGTCGGGGTTGTAATTGG | | |  |  |  |  |
|  |  |  |  |  |  |  |  |
| RCASBPA F | CTGAGCTGACTCTGCTGGTG | | |  |  |  |  |
| Mycn (T&S mut) R | CGCCAACGGGGGCGCGGGCAG | | |  |  |  |  |
|  |  |  |  |  |  |  |  |
| Fgfr2ex18 | GGATTGCTGGCATGCTGTACC | | |  |  |  |  |
| in18R | CCCTGACTGAAAAGGAGCAC | | |  |  |  |  |
